# Can *Trichoderma* Spp. Contribute to the Bioremediation and Biostimulation of Plants in Soil Contaminated with Herbicides?

**DOI:** 10.1021/acsomega.4c09197

**Published:** 2025-01-06

**Authors:** Elaine Damiani Conte, Elton José da Rosa, Gabriel Rieth Silvestrini, Daiane da Silva Motta, Christiane Fernandes de Oliveira, Carine Cocco, Gabriel Fernandes Pauletti, Wendel Paulo Silvestre, Taísa Dal Magro, Joséli Schwambach

**Affiliations:** 1 Laboratory of Biological Control of Plant Disease and Laboratory of Plant Biotechnology, Institute of Biotechnology, University of Caxias do Sul, Rua Francisco Getúlio Vargas, 1130, Petrópolis, Caxias do Sul, Rio Grande do Sul 95070-560, Brazil; 2 University Campus of Vacaria, University of Caxias do Sul, Av. Dom Frei Cândido Maria Bampi, 2800, Barcellos, Vacaria, Rio Grande do Sul 95200-000, Brazil; 3 University of Texas Rio Grande Valley, 1201 West University Dr., Edinburg, Texas 78539- 2909, United States; 4 Laboratory for Studies of the Plant-Environement System and Postgraduate Program in Process Engineering and Technologies (PGEPROTEC), University of Caxias do Sul − Rua Francisco Getúlio Vargas, 1130, Petrópolis, Caxias do Sul, Rio Grande do Sul 95070-560, Brazil

## Abstract

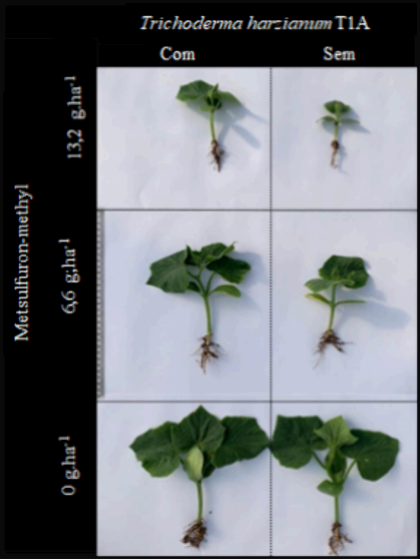

This work aimed to evaluate the potential of *Trichoderma* spp. in the bioremediation of herbicides and biostimulation of plants in herbicide-contaminated soils. In the first phase, the experiment followed a completely randomized design in a 4 × 3 × 4 factorial scheme with five replications, four strains of *Trichoderma* spp., three herbicides, and four herbicide doses. The mycelial growth speed index (MGSI) of *Trichoderma* spp. was obtained by growing it in a Petri dish with PDA associated with different doses of herbicides and evaluated every 24 h for 10 days. Results indicated the selection of*Trichoderma harzianum*T1A to continue the experiment, as it showed an increase in MGSI with metsulfuron-methyl herbicide. The herbicides indaziflam and atrazine reduced the development of *Trichoderma* spp. The second phase evaluated cucumber cultivated at the scheme corresponding to three doses of the herbicide metsulfuron-methyl and with and without application of*T. harzianum*T1A in six pots containing two plants per pot, totaling 12 plants/replications per treatment. The parameters chlorophyll a, chlorophyll b, chlorophyll total index, plant height, phytotoxicity, leaf area index, aerial part dry mass, root length, and root dry mass was assessed in cucumber plants. Herbicide application caused phytotoxicity in cucumber plants and reduced all development parameters evaluated in the first cultivation. The phytotoxic effect was still observed in the second cultivation, leading to reduced root and shoot dry mass using metsulfuron-methyl. The results showed that*T. harzianum*T1A resulted in significant beneficial effects on cucumber development, increasing plant height by 36%, leaf area index by 71%, root length by 23%, shoot dry mass by 54%, and root dry mass by 21% in the first crop, across all herbicide doses used. In the second cultivation, no significant effect of *Trichoderma* application was observed. Therefore,*T. harzianum*T1A contributes to the biostimulation of plants in soil contaminated with metsulfuron-methyl and may contribute to bioremediation.

## Highlights

The herbicides indaziflam and atrazine exhibited suppressive effects on the development of all strains of *Trichoderma* spp.

Metsulfuron-methyl herbicide increased the MGSI of *Trichoderma harzianum* T1A.

*Trichoderma harzianum* T1A application resulted in beneficial effects on cucumber development even in soil contaminated with metsulfuron-methyl.

*Trichoderma harzianum* T1A contributes to the biostimulation of plants in soil contaminated with metsulfuron-methyl and may contribute to bioremediation.

## 1 Introduction

The growing demand for food to meet the needs of the world’s population, combined with the effects of climate change, has led to an increase in the use of pesticides, with estimates of their increased production and global application. According to Tostado and Bollmohr,^1^ the world’s top 10 pesticide-consuming countries are China, the United States, Argentina, Thailand, Brazil, Italy, France, Canada, Japan, and India. In 2020, the United States led with 407.8 thousand tons of pesticides, followed by Brazil with 377.2 thousand tons. However, inappropriate use and handling of pesticides and their behavior in the environment lead to environmental pollution,^2^ requiring strategies to minimize its impact on soil and water and aiming at the environmental sustainability of agricultural systems.^3^

The persistence of pesticides in the soil is closely linked to pesticide contamination in groundwater. Residual herbicides, with their extended period of activity, can inflict a negative environmental impact.^4^ Some herbicides cause phytotoxicity for the subsequent crop due to their long residual period,^5^ requiring a safety interval between application and sowing.^6^ Metsulfuron-methyl is a persistent herbicide in the soil,^7^ and the extensive use of sulfonylurea herbicides can result in environmental contamination.^8^ Atrazine is one of the ten most widely used pesticides in Brazil^9^ and has a high potential to contaminate surface and groundwater.^10^ Indaziflam, on the other hand, is a new pre-emergence herbicide for controlling annual grasses and broadleaf weeds in various cropping systems.^11^ However, studies on the mobility, degradation, and toxicity of indaziflam should be conducted under different scenarios, as there is limited published information available.^12^

Fungi of the genus *Trichoderma* sp. develop well in the soil as they resist various toxic combinations, including herbicides.^13^ They are mainly recognized for controlling plant diseases,^14−18^ inducing disease resistance,^19,20^ and plant biostimulation.^21^ In this sense, research has focused on evaluating the sensitivity and compatibility of *Trichoderma* sp. to various herbicides, aiming to enhance their efficiency in biological control. The findings indicate that different isolates exhibit distinct responses to herbicides.^22,23^

On the other hand, the possible effects of *Trichoderma* sp. for herbicide bioremediation and plant biostimulation, in the sense of improving the quality of contaminated soils, are scarcer. Bernat et al.^24^ revealed that applying *T*. *harzianum* improved the wheat growth stressed with the 2,4-D herbicide and alleviated its toxic effects. At the same time, Tripathi et al.^25^ found *Trichoderma* sp. dominant in the soil region contaminated with heavy metals and herbicides, promoting plant growth. *T. viride* proved to be the most efficient in degrading chlorpyrifos and photodieldrin.^26,27^ Katayama and Matsumura^28^ concluded that *T*. *harzianum* has excellent potential for pesticide degradation, as they verified its ability to degrade various insecticides, including dichloro-diphenyl-trichloroethane and dieldrin. Additionally, *T. atroviride* was noted degrading 2,2-dichlorovinyl dimethyl phosphate (DDVP),^29^ and *T*. *asperellum* holds potential for bioremediation of soils contaminated by polycyclic aromatic hydrocarbons.^30^ Regarding herbicides, Vazquez and Bianchinotti^31^ and Vázquez et al.^32^ reported that certain *Trichoderma harzianum* strains are promising for improving metsulfuron-methyl contaminated soils.

Since herbicides encompass a wide range of chemical families, there is an opportunity to assess the impact of *Trichoderma* sp. on various other molecules. Furthermore, considering the bioremediation of herbicides in the soil as a vital approach for decontaminating contaminated areas and its role in crop management during successive plantings, this work aimed to evaluate the potential of *Trichoderma* sp. for herbicide bioremediation in soil and the biostimulation of plants in herbicide-contaminated soils.

## 2 Material and Methods

This research comprised two phases. First, a selection process identified the best strain(s) of *Trichoderma*, considering survival and *in vitro* development when associated with herbicides, selected for their residual action in the soil and use in areas of large crops (grains and fruit growing). Subsequently, soil tests were carried out in a second phase to evaluate the effect of the selected strain and herbicide.

### 2.1 Effect of Herbicides on the Mycelial Growth of Trichoderma Spp

In the first phase, the experiment was conducted using a completely randomized design in a 4 × 3 × 4 factorial scheme with five replications (each replication corresponding to one plate). The factors included four strains of *Trichoderma* spp. selected from previous studies (*T*. *harzianum* T1A,^33^ and isolates T3, T17, and T19^34^), three herbicides (metsulfuron-methyl, indaziflam, and atrazine) and four herbicide doses. Doses of zero, 50%, 100%, and 200% of the commercial recommendation were used in the package insert for metsulfuron-methyl and indaziflam, marketed under trade names Ally and Alion, respectively. For atrazine (Atrazine), doses of zero, 50%, 100%, and 150% were selected, as 200% would exceed the dilution capacity defined in the assay (Table 1).

**Table 1:** **–** Herbicides and Doses Used in the Experiment^a^

Active ingredient	Commercial product	Active ingredient concentration	Active ingredient dose (g·ha^–1^)	Recommended dose (%)
Metsulfuron-methyl	Ally	600 g·kg^–1^	2, 4, 8	0, 50, 100, 200
Indaziflam	Alion	500 g·L^–1^	38, 75, 150	0, 50, 100, 200
Atrazine	Atrazina	500 g·L^–1^	1625, 3250, 4875	0, 50, 100, 150

a Caxias Do Sul, RS

Strains of *Trichoderma* spp. were preserved in a potato-dextrose-agar (PDA) medium in the fungal collection of the Laboratory of Biological Control of Plant Disease, University of Caxias do Sul. The initial multiplication of the *Trichoderma* spp. was carried out in Petri dishes with PDA medium, placed in a culture chamber with a 12 h photoperiod and a temperature of 25 °C for 15 days. The PDA medium for the experiment was autoclaved and melted under aseptic conditions.

Individual herbicides were dissolved and diluted in aliquots of PDA melting medium. For metsulfuron-methyl herbicide, 0.0132, 0.264, and 0.528 g were weighed, corresponding to 50%, 100%, and 200% of the dose, respectively, of the commercial product, which were then diluted in 5 mL of distilled water. For the herbicide indaziflam, 300 μL, 600 μL, and 1200 μL were used, corresponding to 50%, 100%, and 200% of the commercial product’s dose, respectively, with distilled water added to adjust the volume to 5 mL for each treatment. These doses were poured into an Erlenmeyer flask containing 395 mL of PDA melting medium (40 °C), resulting in 400 mL for each treatment, with 20 mL distributed into each Petri dish. For atrazine, 13 mL, 26 mL, and 39 mL were pipetted, corresponding to 50%, 100%, and 150% of the dose, respectively, of the commercial product, and 27 mL, 14 mL, and 1 mL, respectively, of distilled water were added, resulting in a total volume of 40 mL. These mixtures were subsequently added to the 360 mL of PDA melting medium, yielding 400 mL.

For the control treatments, a volume of distilled and autoclaved water equal to that added to the herbicide treatments was applied to maintain the same dilution of the PDA medium, 5 mL for the herbicides metsulfuron-methyl and indaziflam and 40 mL for atrazine.

In the center of each prepared plate, a 5 mm plug of the *Trichoderma* spp. strain was applied, and then the Petri dishes were kept in a culture chamber at 25 °C and a 12 h photoperiod.

Orthogonal measurements of the diameter (mm) of the fungus colonies were measured daily for 10 days. The data were used to calculate the mycelial growth speed index (MGSI) according to the formula:
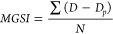
Where: MGSI = mycelial growth speed index;

D = average colony diameter from the present day (cm);

Dp = average colony diameter of the previous day (cm);

N = number of days after inoculation.

MGSI data were submitted for analysis of variance (*p* < 0.05), and the effect of doses of different herbicides on strains of *Trichoderma* spp. was subjected to regression analysis. The equation with significant coefficients (*p* < 0.05) and with the highest correlation (r^2^) was adopted.

### 2.2 Effect of *Trichoderma harzianum* T1A on the Development of Cucumber Plants Submitted to Metsulfuron-methyl Applied in the Soil

For the second phase, the experiment was performed with the *Trichoderma* strain that increased MGSI with at least one of the tested herbicides. Thus, a controlled experiment was carried out in a protected environment on the Campus UCS – Vacaria to evaluate the soil degradation behavior of the metsulfuron-methyl herbicide, both with and without *Trichoderma harzianum* T1A. The experiment was conducted in pots cultivated with cucumber, which acts as a bioindicator species for herbicide residue.

The design employed in the treatments was a completely randomized 3 × 2 factorial scheme, with six replications (each containing two plants per pot). The factors tested included three doses of metsulfuron-methyl herbicide (zero, 4 g·ha^–1^, and 8 g·ha^–1^ of the active ingredient, corresponding to zero, 100%, and 200% of the commercial dose of 6.6 g·ha^–1^ of the commercial product Ally) and with and without the application of *Trichoderma harzianum* T1A. The pots, with a capacity of 8 L, were filled with soil classified as a Hapludox according to Soil Survey Staff,^35^ or *Latossolo Bruno Alumínico típico*, according to Brazilian Soil Classification,^36^ previously used in tomato cultivation and collected at a depth of 0–20 cm. The soil was kept dry in an oven for approximately 12 months, and no fertilization was performed on the cucumber crop.

The herbicide was applied prior to planting the crop using a backpack sprayer pressurized with CO_2_, operating at a constant pressure of 2.0 kgf·cm^–2^. The sprayer was equipped with a bar containing two 110.015 spray nozzles, spaced 0.50 m from each other and 0.50 m from the target and with a speed of 3.6 km·h^–1^. Seven days after this application, 1.5 g of biological powder containing the fungus *Trichoderma harzianum* T1A propagated in rice^37^ and containing 3.5 × 10^9^ conidia·g^–1^ quantified according to the methodology described by Embrapa^38^ was applied to each pot. Five cucumber seeds (hybrid Feisty F1 – Tsv seeds) were sown per pot as a bioindicator plant on the eighth day after the *Trichoderma* application. After emergence, only two plants were maintained in each pot. Manual irrigation was performed every 3 days with a volume corresponding to precipitation of 10 mm.

At 30 and 45 days after cucumber sowing, the plants were evaluated for leaf chlorophyll a, chlorophyll b, chlorophyll total index, and plant height. At 45 days after cucumber sowing, phytotoxicity and the parameters root length, leaf area index, and root and shoot dry mass were evaluated.

Chlorophyll a, b, and total index were determined using a chlorophyll meter (ClorofiLOG - Falker) on each plant’s leaf blade central part per treatment. Plant height was assessed by measuring the height between the ground level and the plant’s apex using a graduated ruler, and the data was expressed in centimeters. A visual evaluation was conducted to determine the symptoms of phytotoxicity caused by metsulfuron-methyl, assigning zero to treatments with no phytotoxicity and one hundred (100%) to complete plant death.^39^ The symptoms of phytotoxicity to the herbicide evaluated included foliar yellowing in the upper and lower portions of the plant, wrinkled leaves, and plants with reduced height.

For root length, the soil was moistened to remove the plants. Measurements were taken from insertion into the soil to the tip of the primary root using a graduated ruler, and the data was expressed in centimeters. Leaves were removed and passed through a CI-203 CID Bio Science laser leaf area measurer to evaluate the leaf area index. Afterward, the aerial part and the root system were placed in a ventilation oven at a temperature of 65 °C until reaching constant mass to determine the dry mass of the root and aerial part, using a precision scale (0.0001 g), with the data expressed in grams.

Additionally, soil samples were collected from treatments with the highest dose of metsulfuron-methyl herbicide, both with and without *Trichoderma harzianum* T1A, with three replicates per treatment. Two samples were taken from the pots’ 0–5 cm layer and homogenized to compose one sample. Soil samples were kept at room temperature and sent to the Pesticide Residues Analysis Laboratory at the Federal University of Santa Maria - RS (LARP-UFSM) for analysis of metsulfuron-methyl residue by Liquid Chromatography coupled to Serial Mass Spectrometry (LC-MS/MS).

On the same day of the collection of plants for evaluation of the first crop (45 days after sowing), the cucumber crop was resown, and all the procedures of the first crop were repeated, except for the application of treatments.

### 2.3 Statistical Analyzes

Data were analyzed for normality using the Shapiro-Wilk test. The interaction between herbicide and *Trichoderma* sp. was evaluated according to two-way ANOVA. In the case of data normality, the comparison of means was performed using analysis of variance (ANOVA) and Tukey’s test (*p* ≤ 0.05) for the comparison between treatments with herbicides and by the *t* test in the comparison of the use of *Trichoderma*. In the case of non-normality, the variables were analyzed using the Kruskal–Wallis test (*p* ≤ 0.05) in the comparison between treatments with herbicide and by the U-Mann–Whitney test (*p* ≤ 0.05) in the comparison of the use of *Trichoderma*. All statistical analyzes were performed using SPSS 21.0 software.

## 3 Results

### 3.1 Effect of Herbicides on the Mycelial Growth of Trichoderma spp

**Figure 1:**
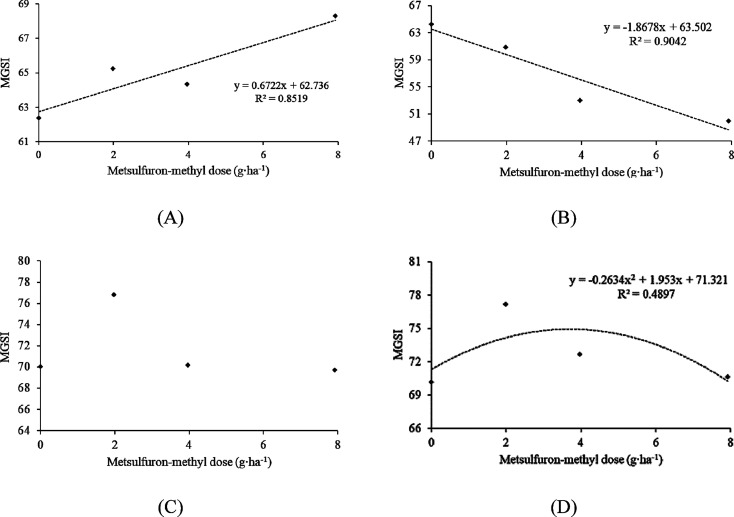
Mycelial growth speed index (MGSI) of four strains of *Trichoderma* spp. at different doses of metsulfuron-methyl herbicide. A: *Trichoderma harzianum* T1A; B: *Trichoderma* sp. T3; C: *Trichoderma* sp. T17; D: *Trichoderma* sp. T19. Graphs with a regression line and equation indicate a significant relationship at a 5% error probability.

The phase I test results demonstrated that the *Trichoderma harzianum* T1A strain presented the MGSI with a positive linear fit for the metsulfuron-methyl herbicide (Figure 1A). The strain of *Trichoderma* sp. T3 (Figure 1B) exhibited a negative linear fit, while *Trichoderma* sp. T19 showed a quadratic fit (Figure 1D), demonstrating that their development was hindered by an increase in metsulfuron-methyl herbicide dose. However, for *Trichoderma* sp. T17 (Figure 1C), there was no significant adjustment of the data to the models for the evaluated conditions and environments, which can be attributed to its compatibility with the herbicide.

**Figure 2:**
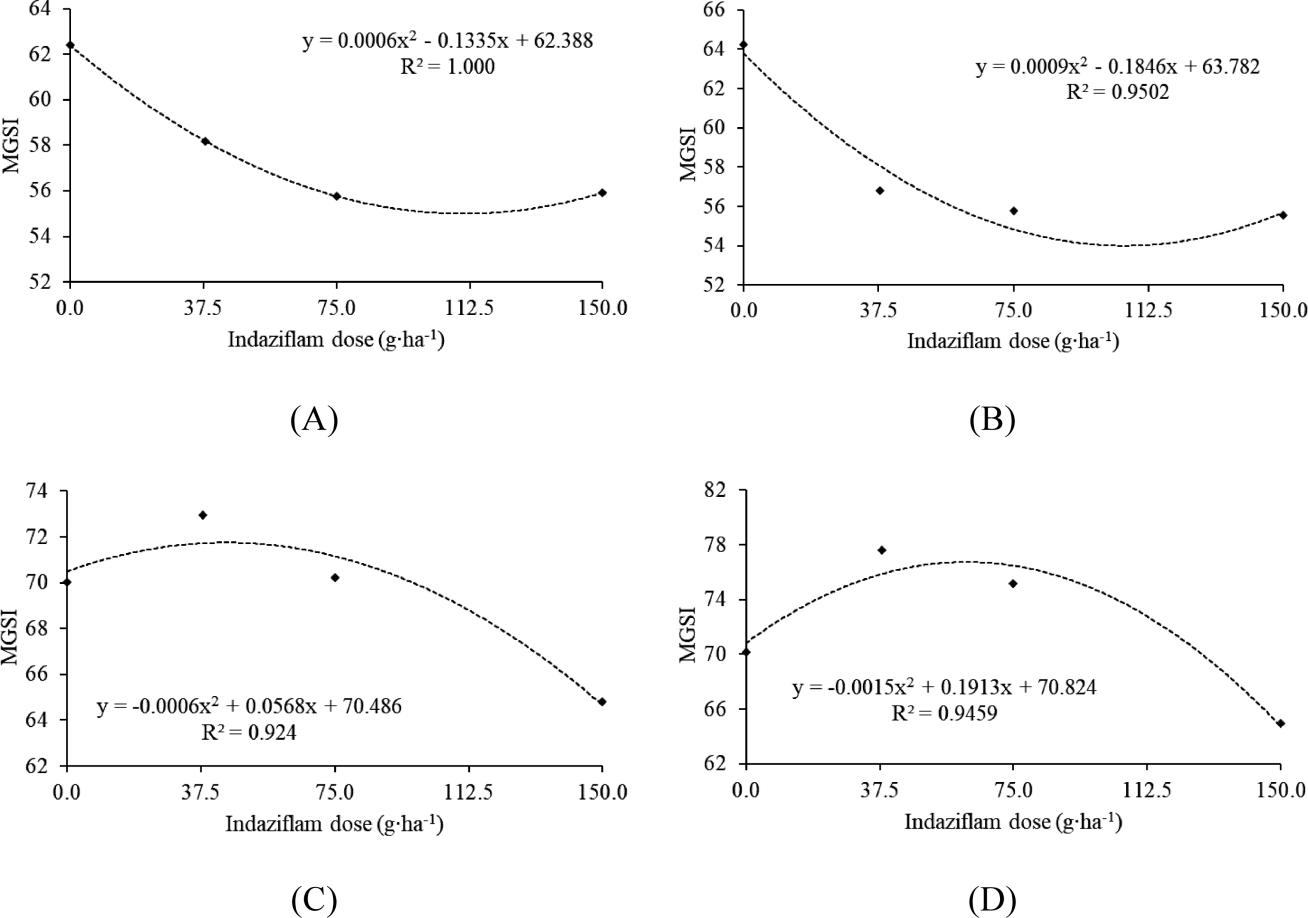
Mycelial growth speed index (MGSI) of four strains of *Trichoderma* spp. in different doses of the herbicide indaziflam. A: *Trichoderma harzianum* T1A; B: *Trichoderma* sp. T3*;* C: *Trichoderma* sp. T17*;* D: *Trichoderma* sp. T19. Graphs with a regression line and equation indicate a significant relationship at a 5% error probability.

In contrast, the herbicide indaziflam reduced MGSI with increasing doses in all *Trichoderma* strains tested, displaying a significant quadratic adjustment (Figure 2 A, B, C, and D). The results emphasize the high toxicity of the herbicide indaziflam on the *Trichoderma* strains tested.

**Figure 3:**
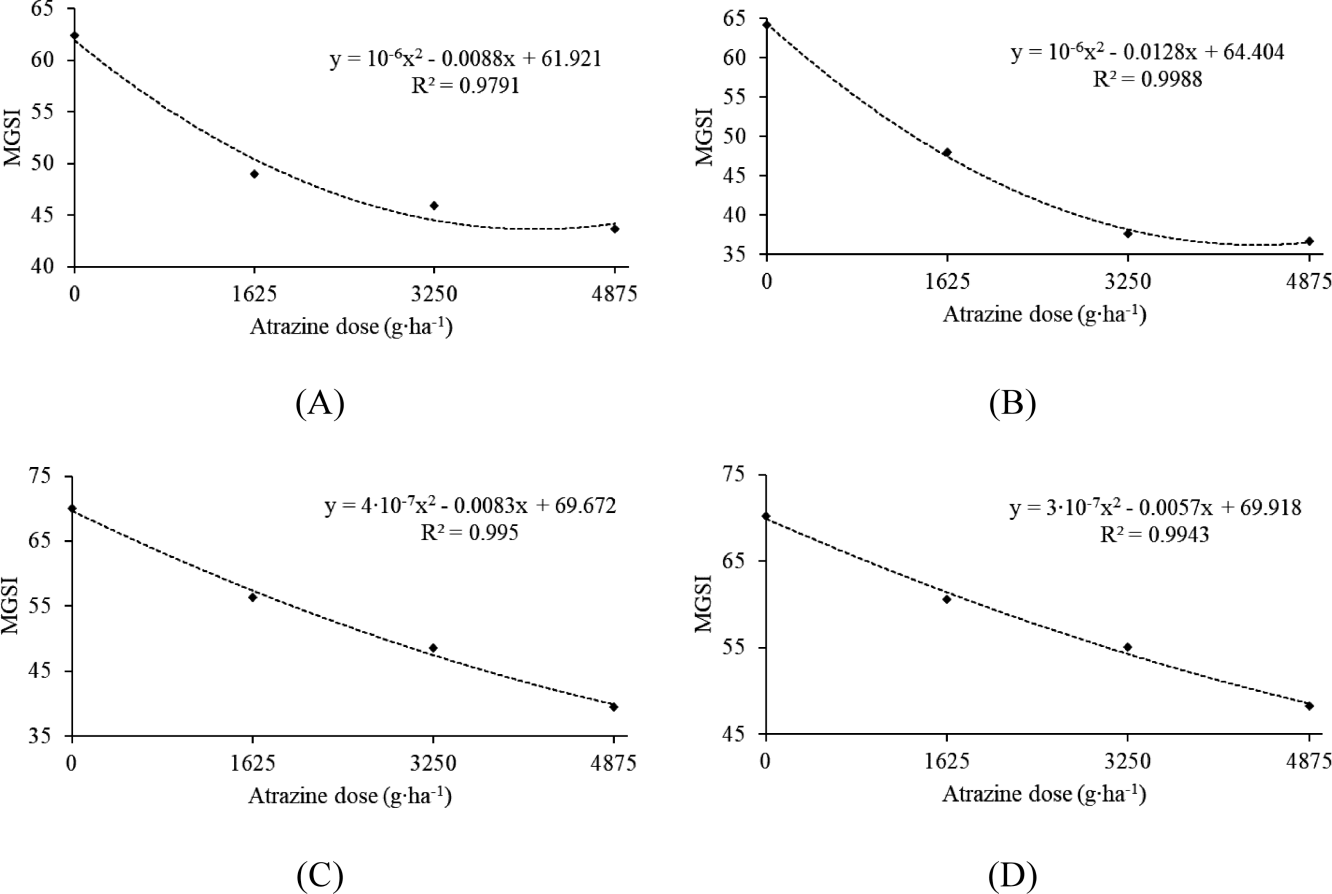
Mycelial growth speed index (MGSI) of four strains of *Trichoderma* spp. in different doses of the herbicide atrazine. A: *Trichoderma harzianum* T1A; B: *Trichoderma* sp. T3*;* C: *Trichoderma* sp. T17*;* D: *Trichoderma* sp. T19. Graphs with a regression line and equation indicate a significant relationship at a 5% error probability.

Regarding the use of the atrazine herbicide, the results showed a significant reduction in the MGSI in all tested doses across the four strains of *Trichoderma*, demonstrating a significant quadratic adjustment (Figure 3 A, B, C, and D). These findings highlight the high toxicity of the herbicide at the tested doses.

In general, the herbicides indaziflam and atrazine exhibited suppressive effects on the development of all *Trichoderma* sp. strains tested. On the other hand, metsulfuron-methyl herbicide increased the *Trichoderma harzianum* T1A development. Consequently, the *Trichoderma harzianum* T1A strain was selected for further testing due to its potential for bioremediation studies in soil contaminated with the herbicide metsulfuron-methyl.

### 3.2 Effect of *Trichoderma harzianum* T1A on the Development of Cucumber Plants Submitted to Metsulfuron-methyl Applied in the Soil

In the second phase of the work, the results did not indicate a significant interaction between the use of *Trichoderma* and metsulfuron-methyl at any of the assessed time points and cultures. Still, the combination of them exhibited notable secondary effects.

In the first crop, after applying the herbicides and the microorganism to the soil, adverse effects on the plants were observed with increasing doses of metsulfuron-methyl (Table 2). Conversely, positive effects on plant height were observed with *Trichoderma* strain T1A at 30-day and 45-day evaluation periods. The chlorophyll a, b, and total indexes increased with the highest dose of herbicide applied in the evaluation at 30 days. At 45 days, higher total and chlorophyll-a indexes were observed in the treatment without herbicide application. Furthermore, at 45 days, there was an increase in total and chlorophyll-a indexes at plots using *Trichoderma*.

**Table 2:** Plant Height and Indexes of Chlorophyll A, B, and Total of Cucumber Plants, Hybrid Feisty F1, at 30 and 45 Days after Sowing, in Pots with Hapludox in the First and Second Cultivation after Application of Metsulfuron-methyl and *Trichoderma harzianum* T1A^a^

	Evaluation at 30 days after sowing				Evaluation at 45 days after sowing			
		Chlorophyll index				Chlorophyll index		
Treatment	Plant height (cm)	a	b	Total	Plant height (cm)	a	b	Total
**First cultivation**								
Metsulfuron-methyl								
Zero	6,8 a	48,0 b	18,4 b	66,3 b	9,0 a	51,5 a	18,5^ns^	70,0 a
6,6 g·ha^–1^	5,1 b	49,4 ab	20,4 b	69,0 ab	8,1 a	47,5 b	16,3	63,8 b
13,2 g·ha^–1^	3,0 c	49,7 a	24,0 a	73,7 a	3,8 b	49,5 ab	18,1	67,6 ab
*Trichoderma harzianum* T1A								
No application	4,7^ns^	49,3^ns^	20,9^ns^	70,3^ns^	6,0 b	48,3 b	16,5^ns^	64,9 b
With application	5,2	48,3	19,2	67,5	8,2 a	50,4 a	18,3	68,7 a
C.V. (%)	39,9	11,8	28,4	14,5	48,1	6,8	17,8	9,1
**Second cultivation**								
Metsulfuron-methyl								
Zero	10,9 a	45,2^ns^	11,8^ns^	57,1^ns^	23,6^ns^	45,5^ns^	15,5 a	61,1 a
6,6 g·ha^–1^	8,8 b	45,7	12,1	57,8	20,3	44,8	14,9 a	59,8 ab
13,2 g·ha^–1^	8,6 b	43,0	12,7	55,7	22,6	44,0	12,3 b	56,3 b
*Trichoderma harzianum* T1A								
No application	9,6^ns^	45,2^ns^	11,6^ns^	56,8^ns^	22,2^ns^	44,4^ns^	14,3^ns^	58,8^ns^
With application	9,1	44,0	12,8	56,9	22,4	45.1	14,1	59,1
C.V. (%)	29,8	13,0	44,8	17,2	29,6	5,2	25,6	10,2

a Vacaria, RS, 2022. Means followed by different letters in the column differ by the Kruskal–Wallis test (*p* ≤ 0.05) for plant height, total chlorophyll at 30 days and chlorophyll b at 30 and 45 days, and by the Tukey test (*p* ≤ 0 0.05) for the other variables in the comparison of herbicide doses and the comparison of the use of *Trichoderma*. The U-Mann–Whitney test (*p* < 0.05) was used for plant height, chlorophyll b, and total at 30 days, and the *t* test (*p* ≤ 0.05) for the other variables. ^ns^ not significant by the F test. C.V.: Coefficient of variation.

After the first cultivation, the analyzed soil samples did not show detectable levels of metsulfuron-methyl herbicide (results were below 0.005 mg.kg^–1^), even at the highest administered dose, and without applying *Trichoderma harzianum* T1A in the soil.

In the second crop, the effects of continued microorganism and herbicide application were evident, with a reduction in chlorophyll b and total indexes evaluated 45 days after sowing. In addition, plant height decreased until 30 days after sowing but showed recovery in the evaluation at 45 days (Table 2).

In the first cultivation, the metsulfuron-methyl herbicide induced phytotoxicity, reducing leaf area, root length, and dry mass of the aerial part and roots. In contrast, using *Trichoderma harzianum* T1A mitigated the herbicide’s toxicity and promoted increased development of the cucumber plants (Table 3). The results obtained in the second cultivation still exhibited a phytotoxic effect, leading to reductions in root and shoot dry mass using metsulfuron-methyl.

**Table 3:** Phytotoxicity caused by the herbicide, leaf area index (LAI), root length, and dry mass of cucumber plants, hybrid Feisty F1, at 45 days after sowing, cultivated in pots with Latosol in the first and second cultivation after metsulfuron-methyl and *Trichoderma harzianum* T1A application^a^

				Dry mass (g)	
Treatment	Phytotoxicity (%)	LAI (cm^2^)	Root length (cm)	Aerial part	Root
**First cultivation**					
Metsulfuron-methyl					
Zero	0 c	130,0 a	5,22 a	0,71 a	0,082 a
6,6 g·ha^–1^	24 b	92,6 a	4,84 a	0,44 b	0,046 b
13,2 g·ha^–1^	66 a	27,2 b	3,39 b	0,15 c	0,022 c
*Trichoderma harzianum* T1A					
No application	40 a	63,2 b	4,06 b	0,35 b	0,046 b
With application	18 b	108,2 a	5,00 a	0,54 a	0,056 a
C.V. (%)	113,3	74,1	35,4	71,9	80,4
**Second cultivation**					
Metsulfuron-methyl					
Zero	0 b	367,3^ns^	6,03^ns^	1,44 a	0,171 a
6,6 g·ha^–1^	10 a	294,1	6,58	1,09 b	0,116 b
13,2 g·ha^–1^	6 ab	348,7	7,19	1,20 ab	0,099 b
*Trichoderma harzianum* T1A					
No application	5,0^ns^	331,3^ns^	7,01^ns^	1,22^ns^	0,131^ns^
With application	4,6	348,1	6,11	1,29	0,128
C.V. (%)	238,8	32,9	29,6	32,5	48,7

a Vacaria, RS, 2022. Means followed by different letters in the column differ by the Kruskal–Wallis test (*p* ≤ 0.05) and by the Tukey test (*p* ≤ 0.05) only for the aerial part dry mass in the second cultivation in the dose comparison of the herbicide and by the U-Mann–Whitney test (*p* ≤ 0.05) in the comparison of the use of *Trichoderma*. ^ns^: not significant by the F test (*p* ≤ 0.05). C.V.: Coefficient of variation.

The reduction in plant development with the use of metsulfuron-methyl stands out, with a decrease of 58% in plant height, 79% in leaf area index (LAI), 35% in root length, 79% in shoot dry mass, and 73% in root dry mass observed at 45 days in the first cultivation with the highest dose applied. Even after 105 days of herbicide application, a decrease in development was still observed in the second cucumber crop’s evaluation at 45 days, with 24% and 42% reductions in the dry mass of the aerial part and roots, respectively.

The use of *Trichoderma harzianum* T1A significantly improved cucumber development, increasing plant height by 36%, LAI by 71%, root length by 23%, shoot dry mass by 54%, and root dry mass by 21% in the first crop across all herbicide doses used. In the second cultivation, no significant effect of *Trichoderma* application was observed. However, the product was also not reapplied in the second cultivation, indicating the possible need for reapplication to improve efficiency.

## 4 Discussion

### 4.1 Impact of Different Residual Herbicides on the Development of Trichoderma spp

The strains chosen to be evaluated in this study were previously studied for their performance as biocontrol agents and in promoting plant growth.^33,34^ Considering that *Trichoderma* sp. is predominantly applied to soil and that the vast majority of agricultural soils are contaminated with pesticide residues^40^ it is crucial to understand the performance of these strains in the presence of herbicides and to assess whether they can contribute to bioremediation and plant biostimulation under such conditions. So, our study focused on the search for a *Trichoderma* strain capable of biostimulating plants in an environment contaminated with herbicides and the potential bioremediation of these herbicides in the soil. The herbicides (metsulfuron-methyl, indaziflam, and atrazine) were selected for their residual action in the soil and usage in areas of large crops (grains and fruit growing). Among the herbicides tested, only metsulfuron-methyl herbicide allowed increased MGSI of *Trichoderma harzianum* T1A. On the other hand, the herbicides indaziflam and atrazine exhibited suppressive effects on the development of all strains of *Trichoderma* spp.

The results revealed that the strain of *Trichoderma harzianum* T1A benefits from the presence of the herbicide metsulfuron-methyl in the culture medium, suggesting its potential for bioremediation studies in soil. The results are consistent with previous research by Vazquez and Bianchinotti^31^ and Vázquez et al.,^32^ who isolated three strains of *Trichoderma harzianum* capable of using metsulfuron-methyl as a carbon and energy source. The authors also examined their *in vitro* detoxification abilities and recommended further studies for soil applications.

According to González-Delgado et al.,^41^ adverse or limited effects of indaziflam on soil biology were not expected due to its mode of action and the absence of chlorine in the molecule. However, they suggested that further studies are needed to assess the potential toxicity of indaziflam under laboratory and field conditions. The herbicide indaziflam did not significantly affect microbial biomass but reduced the fungal colonies in treated soils.^42^ Indaziflam did not negatively affect microorganisms active in soil carbon and nitrogen mineralization.^43^ However, specific tests focused on using the genus *Trichoderma* were not found. Thus, the results obtained in the present experiment indicate the necessity to better evaluate the potential for toxicity in the field at different doses, considering that it significantly and negatively affected the development of the four strains of *Trichoderma*.

Certain strains of *Trichoderma* have already been shown to be resistant to atrazine concentrations of up to 10,000 mg·L^–1^, effectively degrading 89% of atrazine in 40 days.^44^ However, in the present experiment, the lowest dose used corresponded to a concentration of 16,250 mg·L^–1^ (1625 g·ha^–1^ atrazine), which proved to be toxic to the *Trichoderma* strains even at half the recommended dose for field application (recommended dose: 6.5 L·ha^–1^ and syrup volume 100 L·ha^–1^). Nevertheless, under field conditions, the herbicide is distributed in the soil profile, and contact with microorganisms may be less than exposure to the same dose in Petri dishes. Furthermore, the soil protects microorganisms through its clays and organic matter. Despite this, it is crucial to test and select new strains for their capacity to degrade atrazine at different concentrations.

### 4.2 Impacts of *Trichoderma harzianum* T1A on Cucumber Development and Biodegradation in Soil with Metsulfuron-methyl

*Trichoderma harzianum* T1A had beneficial effects on cucumber development even in soil contaminated with metsulfuron-methyl. Chlorophyll content in the leaves is one of the indicators of the level of damage that a given stress may be causing to the plant.^45^ Metsulfuron-methyl application reduced chlorophyll in *Solanum nigrum* Riethmuller-Haage et al·,^46^ similar to this work’s results. On the other hand, the increase in total chlorophyll with the use of *Trichoderma harzianum* T1A, even in plants subjected to the highest dose of metsulfuron-methyl, may indicate that its use reduces plant stress. Conte et al.^47^ also observed a significant increase in the total chlorophyll content in the vegetative stage of the soybean crop with the application of *Trichoderma* spp.

Moreover, the use of *Trichoderma harzianum* T1A proved to be efficient in mitigating phytotoxicity and in biostimulating cucumber plants, even in soils subjected to applications of twice the recommended dose of metsulfuron-methyl (13.2 g·ha^–1^), what could be related to it resistance to or capacity of biodegradation of metsulfuron-methyl, allowing it to promote plant growth. This finding aligns with the work of Vázquez et al.,^32^ who observed *in vitro* that *T*. *harzianum* successfully detoxifies metsulfuron-methyl, with different strains displaying varying degrees of efficiency in herbicide degradation.

The ability of *Trichoderma* spp. to metabolize different types of pesticides has been recently proven.^30,32^ However, the effects of the development of plants in soil observed in the present work have not yet been reported in the literature. Therefore, the biostimulation observed in the present work reinforces the idea of the capacity of some strains of *Trichoderma* to biodegrade metsulfuron-methyl in the soil.

Biodegradation could not be proven with the analyzed soil samples, which did not show detectable levels of metsulfuron-methyl herbicide, even at the highest administered dose, and without applying *Trichoderma* in the soil. According to Silva,^48^ metsulfuron-methyl is employed in minute quantities and is found in the soil in trace concentrations, rendering its detection challenging. Also, a chemical analysis can only partially assess the toxicological impact of contaminants by quantifying their concentration in tested materials such as soil, plants, or water. However, it does not reveal the effects of pollutant levels on biological processes within living organisms, including cellular activities.^49^ Only such insights can provide a comprehensive understanding of the risks associated with a contaminant’s presence in the environment. Therefore, using cucumber as a bioindicator plant is a more efficient practice for evaluating metsulfuron-methyl residue in the soil, as demonstrated in the present work. Silva et al.^50^ also observed cucumber as a bioindicator efficient due to a reduction in the dry matter of the aerial part of cucumber plants with increased herbicide dose, confirming the adverse effects of metsulfuron-methyl on physiological characteristics.

Metsulfuron-methyl, from the sulfonylurea group, stands as one of the most widely used herbicides for postemergence weed control in wheat in southern Brazil^51^ and was identified as a compound posing a critical environmental risk concerning potential contamination of areas and groundwater.^52^ Unfortunately, the toxicity of sulfonylurea has a significant impact on the microorganisms in the soil.^53^ In addition, producers have used this herbicide for early desiccation to diminish the density of difficult-to-control species, such as *Conyza* spp., before soybean sowing.^50^ Notably, using metsulfuron-methyl herbicide in desiccation management necessitates a safety interval between its application and the sowing of sensitive crops. For soybean cultivation, this recommended interval is 60 days.^6^ In this context, using *Trichoderma* could contribute to the bioremediation and biostimulation of plants, mitigating damage to the soybean crop, anticipating the implantation of the crop, and reducing environmental groundwater contamination. However, further studies focusing on the biodegradation of the herbicide with the aid of *Trichoderma* are imperative to establish practical recommendations.

In this study, we demonstrated the effect of *Trichoderma harzianum* T1A in biostimulating plant growth in soil contaminated with metsulfuron-methyl. This procedure can be highly beneficial in developing susceptible crops in contaminated soils. Furthermore, it may contribute to the bioremediation of the herbicide in the soil, requiring further confirmatory experiments. Hence, the use of *Trichoderma* in the soil offers several advantages,^54^ such as controlling different plant diseases, improving plant growth, and fostering a cleaner environment, all of which are beneficial for promoting sustainable agriculture^55^ and potentially bioremediating areas with metsulfuron-methyl residue.

## 5 Conclusions

When evaluating the performance of four *Trichoderma* sp. isolates at different herbicide doses, we found that only *Trichoderma harzianum* T1A could maintain its development and only in the presence of metsulfuron-methyl. When assessing its performance in soil and its interaction with cucumber plants in pots, we determined that *Trichoderma harzianum* T1A contributes to the biostimulation of plants in soil contaminated with metsulfuron-methyl and may contribute to bioremediation. Future field studies should help better understand this isolate’s role in plant biostimulation in contaminated soils.
